# Ethnopharmacological Study of the Medicinal Plants Used in the Treatment of Sickle Cell Anemia in the West Region of Cameroon

**DOI:** 10.1155/2022/5098428

**Published:** 2022-04-26

**Authors:** Natacha Lena Yembeau, Prosper Cabral Biapa Nya, Constant Anatole Pieme, Kevin Dedjam Tchouane, Christian Bernard Kengne Fotsing, Prudence Josela Nya Nkwikeu, Alfloditte Flore Feudjio, Phelix Bruno Telefo

**Affiliations:** ^1^Research Unit of Biochemistry of Medicinal Plants, Food Sciences and Nutrition, Department of Biochemistry, Faculty of Science, University of Dschang, P. O Box 67, Dschang, Cameroon; ^2^Laboratory of Biochemistry, Department of Biochemistry and Physiological Sciences, Faculty of Medicine and Biomedical Science, University of Yaounde I, P. O Box 1364, Yaounde, Cameroon

## Abstract

**Background:**

Sickle cell anemia (SCA) or sickle cell disease (SCD) is a genetic disease associated with increased morbidity and mortality in Africa and other developing nations. Therefore, modern and traditional remedies are being introduced for use in the treatment and management of this disease. This is because safe, effective, and inexpensive therapeutic agents are urgently needed for the treatment of this disease in Africa and other developing nations.

**Objective:**

The purpose of this study is to identify medicinal plant species commonly used by traditional healers in the treatment of sickle cell patients across some localities in the west region of Cameroon. *Material and Methods*. The ethnopharmacological survey was carried out in several districts within some localities of the western region of Cameroon. The survey was based on a semistructured questionnaire that was administered to 17 traditional healers and 62 sickle cell patients. It took place between November 2018 and March 2019. Personal information of participants and plant therapy data were gathered. Plants were identified at the National Herbarium of Cameroon. Literature review determined pharmacological effects and phytochemical compounds of the identified plants. Data were generally analysed using Epi Info 7 software for Windows.

**Results:**

Twelve medicinal plant species belonging to 10 families are being used in the treatment of sickle cell anemia across the study sites. Euphorbiaceae is the dominant family with three plant species. Bark (39.3%) and seeds (35.7%) are the most used plant parts, which get administered through maceration, decoction, and chewing in water. According to the literature review, the identified plants have pharmacological effects and phytochemical compounds (especially polyphenols and alkaloids) that signify the presence of antioxidant compounds, which may possess an antisickling activity. There is therefore a need to conduct another study to scientifically validate (in vitro) antisickling properties of these plants.

**Conclusion:**

This study has revealed promising medicinal plants that are currently applied in the traditional treatment of sickle cell anemia. Although still inconclusive, the association of pharmacological effects and phytochemical compounds with these medicinal plants justifies their use in traditional pharmacopoeia.

## 1. Background

The sickle cell anemia (SCA), sickle cell disease (SCD), or drepanocytosis is a life-long blood disorder characterized by red blood cells that assume an abnormal, rigid, and sickle shape. It is one of the most common hemoglobin abnormalities that cause morbidity and mortality, particularly in Africa and the world in general. The SCA is a genetic disorder characterized by the presence in red blood cells of hemoglobin S (Hb S), an abnormal form of the protein used to fix and transport oxygen [1]. It is attributed to a unique mutation of the globin beta gene located on chromosome 11, resulting in the replacement of glutamic acid (Glu) present in hemoglobin A (Hb A) by valine (val) in Hb S, which results in a total change in the spatial conformation of Hb [2]. The physiopathological pattern of sickle cell anemia is based on the fact that, at low oxygen pressure, deoxy-HbS polymerizes and is organized into large fibers inside the red blood cell, which deform and weaken it [2]. It is a particularly common disease in people from Sub-Saharan Africa, India, and Mediterranean countries. It is the most widespread genetic disease in the world, affecting more than 50 million people, including 38 million in Sub-Saharan Africa [3]. The prevalence of SCD can be objectively determined by calculating the birth prevalence of affected children, which requires accurate diagnosis and registration at birth. Approximately 300,000 children are born every year with SCD in the world, and countries such as the United States of America, United Kingdom, and Jamaica have well-documented SCD population. However, this SCD population constitutes only 1% of the global population of SCD, as over 75% are in Sub-Saharan Africa [4]. In recent years, bone marrow transplantation and gene therapy have been proposed as an efficient way of treating SCD. However, the cost implications, availability of necessary expertise, problem of finding suitable donors, inadequate transfusion, and transfusion-related infections constituted a major setback to this approach in developing countries [1]. The management of the disease is ensured by the establishment of many treatments based mainly on extracts containing antioxidant activities. Hydroxyurea achieved this function by activating the production of fetal hemoglobin to replace the hemoglobin S that causes sickle cell anemia. One of the mechanisms for the action is based on its ability to inhibit the reaction that leads to the production of deoxyribonucleotides by acting on the enzyme of ribonucleotide reductase. The production of deoxyribonucleotides requires tyrosyl group (which is a free radical). So, hydroxyurea captures these tyrosyl free radicals thereby preventing the production of deoxyribonucleotides. Another mechanism is that it increases nitric oxide levels. This brings about the activation of soluble guanylyl cyclase, which results in an increase in the cyclic GMP. It also activates gamma-globulin synthesis, which is required for the production of fetal hemoglobin [5]. Many drugs those are available for treating the disease are insufficiently effective, toxic, or too expensive. Furthermore, advanced medical care for people suffering from sickle cell anemia (especially in low- and middle-income countries) is also limited by the inaccessibility of healthcare centers. This situation is partly attributed to poverty, socioeconomic, and cultural contexts [6]. According to literature, numerous medicinal plant species across the developing and developed nations have proven to be efficacious in and have high potential due to the presence of antisickling activity for the treatment and management of SCA or SCD. Indeed, using a semistructured questionnaire, Famojuro and Moody [7] conducted an ethnobotanical study on the plants generally used in the management of the disease by the populations of the south-west region of Nigeria. This survey revealed that the 44 collected plant species have high potential for drug discovery for disease management. Ismaila [8] showed the antisickling potential of aqueous extracts of three plants: *Cajanus cajan* leaf and seed, *Zanthoxylum zanthoxyloides* leaf, and *Carica* papaya, revealing that these extracts possessed numerous secondary metabolites (tannins, saponins, alkaloids, flavonoids, and glycosides) which could be used as an antisickness treatment alternative to hydroxyurea for disease management. In addition, Munganda [9] identified the plants of the city of Kitty in the Democratic Republic of Congo with 26 traditional healers and conducted studies on the in vitro pharmacological activities of these plants; their study revealed that 80 percent of the plants collected possessed interesting antisickling activities and justified their use in traditional medicine. All these prove that medicinal plants are invaluable resources for local communities and the pharmaceutical industry. Therefore, their use requires ethnobotanical surveys that allow drawing up a nonexhaustive list of promising plant species used in traditional medicine [10]. The knowledge on indigenous medicinal plants is often accompanied by multiple uses and preparations, indications, and dosages for the diseases and symptoms that can be treated. Indeed, the art of healing with medicinal plants has been known and practiced in Africa and elsewhere for time immemorial. This practice usually exploits knowledge transmitted orally from one generation to another through initiated individuals such as traditional healers and herbalists [11]. Practices in traditional medicines vary widely from one country/region to another. They are influenced by factors such as culture, history, and personal philosophy. The Cameroonian flora is full of a large reserve of edible, ornamental, and medical plants. This flora contains more than 8,500 plant species [12]. All these various plants could not be of significant value populations of the world if the information regarding their use has been kept for use only by traditional healers or certain populations. The discovery of contemporary drugs from ingenious medicinal plants is generally based on ethnomedicinal studies [13, 14]. Therefore, documentation of traditional (indigenous) knowledge on use of medicinal plants to treat various diseases is extremely important as a basis for the development of new vital drugs [15]. In modern pharmacopeia, it is well known that 25% of drugs come from plant-based medicinal products. Additionally, chemicals isolated from plants are used to manufacture several synthetic drugs [16]. Therefore, the purpose of this study was to identify medicinal plant species commonly used by traditional healers in the treatment of patients suffering sickle cell anemia across some localities in the west region of Cameroon.

## 2. Material and Methods

### 2.1. Study Site

**Figure 1:**
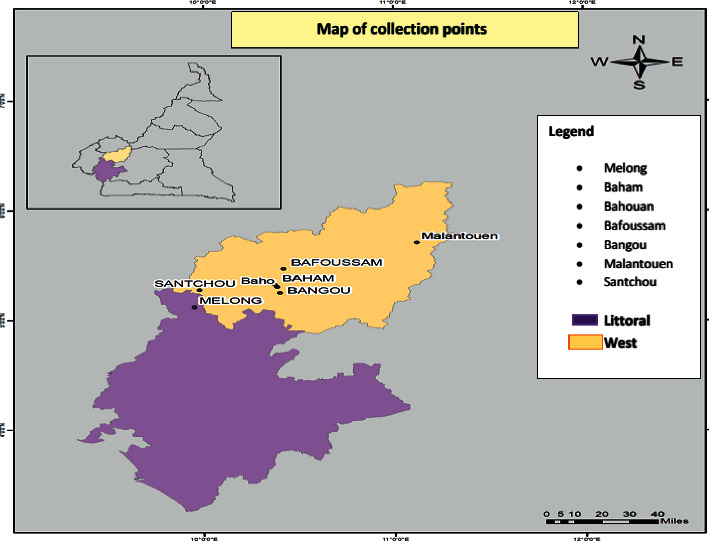
Location of the study area in the western region of Cameroon (made by Mr. Modiko Tony in 2019).

The ethnobotanical survey was carried out in several districts in some localities of the western region of Cameroon, which are Foumban, Bangou, Dschang, Bafoussam, Baham, and Bandjoun, as shown in Figure 1.

Bafoussam is a city in Cameroon, the capital of the western region. It has an estimated population of 347,517 inhabitants in a total area of 402 km^2^ (with urban area of 91 km^2^). Its weather comprises an average temperature of 24°C, north easterly wind blowing at the speed of 11 km/h and 56% humidity. Currently, the city has 41 (forty-one) villages (neighborhoods), each headed by a third degree chief, of which 22 (twenty-two) are for urban spaces and 19 (nineteen) for rural areas. The soil of Bafoussam I is in places lateritic; however, there are also areas of deep brown soil. In the shallows and the edges of rivers, hydromorphic soils are found. Overall, the soil consists mainly of metamorphic rocks, covered in places by volcanic ash (Noun and Njingah areas). On the side of Ndiembou, Ndiengso, and Banengo, we find a lateritic red ferralitic soil. Hydromorphic soils are found in lowlands and along streams. Although not very fertile in certain places, the soil in the communal area is favorable to agricultural activities, the little rugged relief presents flat areas and hills. Due to the existence of dense vegetation in the communal area, the fauna is mainly made up of many birds and small rodents (rats, hedgehogs, porcupines, etc.) whose presence is very notable during the cultivation period.

Foumban city covers an estimated area of approximately 418 km^2^ with a population of 152,728 inhabitants with the Sudano-Guinean climate and two seasons, i.e., a rainy season (mid-March to mid-November) and a dry season (mid-November to mid-March). The annual rainfall amounts sometimes exceed 1700 mm. Temperatures vary between 18 and 23°C. Agricultural and pastoral activities are permanently practiced annually. The relief on the whole territory of the Foumban municipality is dominated by plains, plateaus, and mountains or hills. The forest reserve covers an area of 2100 ha. Incomes or livelihoods are generally derived from agricultural, livestock, or craft activities. The ethnopharmacological survey was carried out in two districts of the city of Foumban, namely, Malantouen and Massagam.

Dschang is a historic and university town in Cameroon located in the western region, in Bamiloric country. It is the second largest city in the region after Bafoussam and ahead of Foumban, Mbouda, and Bangangté. It covers an estimated area of 225 km^2^ with an altitude of 1380 m ASL (above sea level) and the population of 76,524 inhabitants. The city of Dschang is mainly constituted of Bamileke ethnic group (in majority), Hausa, whose imprint is visible on the urban space by the existence of the Hausa quarter, the Bamouns, and the Mbo. Many of these groups are made up of students, hence the observation at the end of the academic year of the so-called “girl's day” festivities. It is a real interethnic harmony in a cultural fair which is lived through sports meetings, promotion of traditional dishes, and dances. Regarding the activities generally carried out, one can speak of a real national integration at this level where each ethnic group is deployed. The activities in this locality are agriculture and animal rearing.

Bangou and Baham are located in the Hauts Plateaux in the Bamileke group. These localities have easterly winds at speeds of 8 km/h and 11 km/h and 59% and 51% humidity levels, as well as populations of 15,787 and 19,680 inhabitants, respectively.

Bandjoun is the capital of the Koung-Khi Department, a commune located in the western region created as a rural commune in 1959. It was set up as a “commune of Pète-Bandjoun” in 2007. It has an estimated population of 6,872 (2012) which covers an estimated area of 274 km^2^ with an altitude of 1530 m ASL. Its weather comprises an average temperature of 25°C and easterly winds at speeds of 11 km/h and 51% humidity. The soils of the highlands, derived from volcanic rocks, are rich and favorable to industrial crops (coffee). The hydromorphic soils of the Noun valley are very rich and can be used for intensive cultivation of food products for local consumption or for export. Its climate is tropical in altitude with two main seasons, one dry and the other rainy. Temperatures are generally low and cool whilst rains are abundant.

The equipment used to collect samples included pruning shears, machetes, newsprint, and paperweight to facilitate the dryness of samples; a digital camera for some pictures of plants; and survey sheets to collect information about plants.

### 2.2. Ethnopharmacological Survey of Medicinal Plants

At the beginning of our investigation, we approached the president of the traditional healers in the western region who had a classified file containing the names and contacts of the various traditional healers treating various diseases, including sickle cell anemia. Discussions took place between the researchers and the chiefs residing at the study locations. The purpose of such discussions was twofold: to explain to the respective chiefs the intention to undertake the ethnopharmacological survey in those localities and to obtain their consents. The acquisition of information required the help of two indigenous interpreters from the cities of Foumban and Bafoussam. After that, we obtain the consent of populations and the consent of the parents and legal representatives of patients who were not able to answer the various questions. Once consents were granted, the survey was carried out using a questionnaire that was administered to 17 traditional healers and 62 sickle cell patients. Participants, especially the older ones, were selected based on the perception that they had great knowledge of traditional medicinal plants. In the end, the survey was conducted with all participants that accepted to respond and fill in the questionnaire sheets. The survey was conducted between November 2018 and March 2019. All interviewees were first informed of the objectives of the study. Interviews covered personal information and plant therapy data. On the one hand, personal information comprised status, educational level, and age of participants. Plant therapy data, on the other hand, constituted parts used and their state of use, preparation, and administration of remedies (oral and massage), families, common and scientific names, and reference or (voucher) numbers, as well as pharmacological and phytochemical aspects. Local, French, and pidgin languages were used in order to easily facilitate interactions with participants or interviewees.

Fresh parts (stems + leaves + flowers) of the identified medicinal plants were collected and compressed between 2 sheets of paper and dried out in the attic. Then, the plants were identified at the National Herbarium of Cameroon (Yaoundé) where their full scientiﬁc names and voucher numbers were obtained. Moreover, some pictures of each sample were obtained for confirmation of the identified plant species. Relative to the disease itself, the interaction in the form of an interview was done during the monthly meetings of the West Regional Association of Sickle Cell Disease (ALDREO) located in Bafoussam, Cameroon. Moreover, other patients met in the villages out of that association also attended the face-to-face interviews.

### 2.3. Literature Review on Pharmacological and Phytochemical Aspects of Medicinal Plants

Furthermore, literature review was undertaken to validate therapeutic/pharmacological effects and phytochemical compositions of the medicinal plants that were identified during the ethnopharmacological survey (see Subsection 2.2).

### 2.4. Data Analysis

The data obtained were subsequently analysed using Epi Info 7 software for Windows. Such data were presented as percentages in graphs, pie-charts, and histogram. Finally, other data on pharmacological and phytochemical aspects generated from literature review were presented in a table.

## 3. Results

The results obtained revealed that most of the patients as well as the traditional healers used the same plants. A total of 12 plants were collected, transported, and authenticated at the National Herbarium of Cameroon.

### 3.1. Status Ages and Educational Levels

#### 3.1.1. Status Ages

**Figure 2:**
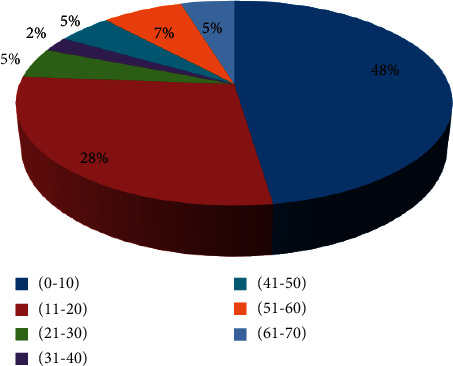
Ages of respondents.

A total of 79 participants were interviewed during the survey, of which 17 and 62 were traditional healers and sickle cell patients, respectively. During the investigation, all traditional healers were found to be male; this was due to the fact that in this western region, only male traditional practitioners were listed as treating sickle cell disease. The age of the participants varied from 1 to 70 years. The most represented age group was that of 1 to 10 years old (48%) represented by their legal representative, followed by that of 11 to 20 years old (28%), while the remaining age five groups (especially between 20 and 71 years) were by far the least represented (Figure 2).

#### 3.1.2. Educational Level of Participants

**Figure 3:**
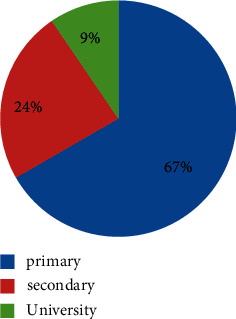
Education levels of participants.

Participants were also categorized according to their level of study. Results in Figure 3 showed that 66.7% (majority of participants), 23.8%, and 9.5% (the least participants) acquired primary, secondary, and university levels of education, respectively.

### 3.2. Parts and State of the Plant Used

**Figure 4:**
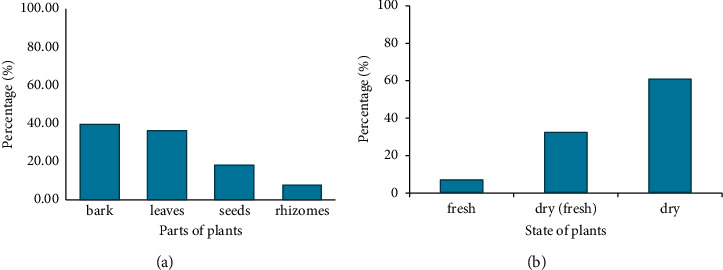
Parts (a) and state (b) of plants used.

Figure 4(a) presents the following plant parts that were used for diverse preparations of treatments: bark (39.29%), seeds (35.71%), leaves (17.86%), and rhizomes (7.14%). The majority and minority of respondents used bark and rhizomes, respectively. Bark extracts were used mostly in Foumban (Malantouen and Massagam) and Baham followed by the leaf extracts that were used in Malantouen, which were the most used parts in the preparation recipes for sickle cell patients. In these localities, plants are usually used in dry (60.7%) and fresh (7.1%) states, with a predominant use of dry state (Figure 4(b)).

### 3.3. Techniques for Preparing Medicinal Remedies

**Figure 5:**
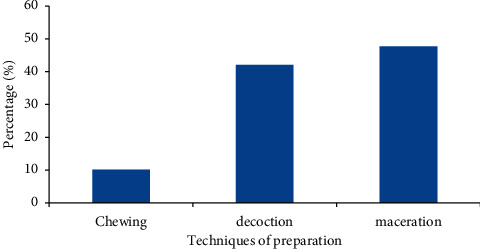
Different preparation methods were used in the studied localities.

Figure 5 presents three techniques that were generally used to prepare remedies by the participants. Those were maceration (47.71%), decoction (42.10%), and chewing (10.19%) in water (Figure 5).

### 3.4. Families of Studied Plants

**Figure 6:**
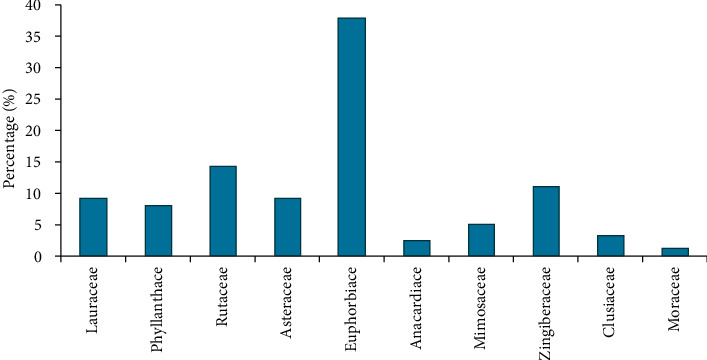
Plant families studied.

During this survey, 10 families of medicinal plants traditionally used in the management or treatment of sickle cell anemia were recorded (Figure 6). The family of Euphorbiaceae is the most represented (37%) with three medicinal species.

### 3.5. Scientific Names of Harvested Plants

Table 1 presents scientific names of the identified 12 medicinal plant species belonging to 10 families as well as reference numbers of and other information about the medicinal plants that were harvested and or used by the respondents. Euphorbiaceae is the only plant family with more than one species (*Jatropha gossypiifolia* Linn., *Jatropha curcas,* and *Ricinodendron heudelotii*).

### 3.6. Pharmacological/Therapeutic Effects and Phytochemical Compositions of Recorded Medicinal Plants as Reported in the Literature

Table 2 presents a summary of pharmacological effects and phytochemical compounds of the recorded medicinal plants in this study. Those plants possess diverse pharmacological potential such as anticancer (6/12), antiparasite (4/12), anti-inflammatory (3/12), antibacterial (3/12), antidiabetic (2/12), antioxidants (4/12), antihypertensive (3/12), and antianaemia (2/12). Although it is not that clear with the majority of other plant species, *Jatropha gossypiifolia* Linn. possesses antianemic activity. Moreover, these plants also contained diversified phytochemical compounds. The most encountered ones were polyphenols (12/12) and alkaloids (11/12) that are, amongst others, sources of antioxidant activity.

## 4. Discussion

Data were derived from ethnopharmacological survey that was based on interviews with 17 traditional healers and 62 sickle cell patients as well as information from the literature review. Findings have revealed that ages of traditional healers were between 40 and 70 years with four whose ages were between 60 and 70 years old. In contrast, the majority of participating patients (62) was youth due to ages largely less than 40 years. However, the traditional healers' profile is similar to that recorded in other studies. This finding confirms that the practice of traditional medicine is generally undertaken by adults, who tend to provide more reliable information [37]. However, the indigenous knowledge on the use of medicinal plants is usually transferred verbally from one generation to another [38]. Despite the willingness of adults to transfer indigenous knowledge, young people are not really interested in the traditional medicinal plants [38]. What is of serious concern is that a large number of participating sickle cell patients are youth, which makes the reliability and authenticity of information gathered from them questionable, also concerning was the relatively large number of participants that had low levels of education (as most possessed primary and secondary) as opposed to the least number of those that had a university qualification.

The study successfully identified a diversity of medicinal plants (12 species and 10 families) that are used in the treatment and management of sickle cell anemia across the study localities in Cameroon. Literature has also revealed many promising plant-based remedies for SCA in countries such as Congo, Nigeria, and Pakistan. This study and the other undertaken in Nigeria have Euphorbiaceae as the most prominent family with three plant species that include *Jatropha gossypiifolia*. This family has also been reported to be predominant in related studies [7]. The occurrence of this family suggests their importance as repository of useful plants which may be explored scientifically in drug development for SCD. These plant species have potential to produce plant-based remedies that have minimal or no side-effects and are easily accessible and affordable for the treatment of sickle cell patients. Parts of the medicinal plants used were bark, seeds, and leaves, with the majority of participants preferring to use bark and seeds. These parts are used in either fresh or dry state, which is predominant. In another study, use of leaves was also preferred to other parts [39]. The variability of plant parts used demonstrates the possibility of gaining a wide variety of biological molecules to justify the therapeutic use of plants [8]. The preference for parts used could be explained by availability, ease of harvesting [40, 41], and the (perceived) abundance of secondary metabolites that are responsible for the biological properties or activities [42]. However, the excessive harvesting of bark and seeds in a long term is likely to be unsustainable. This may lead to local extinction of important medicinal plants and therefore compromise future supplies of the needed remedies. In contrast, any rate of use of leaves has no adverse effect on the life of medicinal plants and is therefore encouraged. Participants use techniques that were generally used by the traditional healers. Medicinal products were consumed in the following forms: maceration, decoction, and chewing in water. Maceration and decoction were predominant modes. During this survey, patients also consumed medicinal plants in liquid form and orally. Decoction is one of the most widely used methods of preparation probably because it allows the collection of the most active ingredients, reduces or cancels the toxic effect of certain recipes, and disinfects the plant [11]. The most used administration or consumption mode was oral route, which can be explained by the fact that metabolites contained in the liquid form are usually assimilated rapidly [1, 43]. In another study, the abovementioned techniques of plant preparation enable the collection of the most active compounds for pharmacological properties [11, 44]. As far as the dose is concerned, participants used various units to estimate the quantity of plants. Those include finger length (bark and stem), pinch (powdered), and numbers (leaves, seeds, and rhizomes) and spoons. The challenge is these methods are likely to result in overdose due to possible imprecision. The lack of precise dosage is one of the drawbacks of traditional medicinal plants [45]. According to another study [31], bark of *Fagara tesmannii,* rhizomes of *Curcuma longa,* and seeds of *Persea americana* have shown a greater inhibitory effect on red blood cell sickling. The study has indicated the in vitro the action of the extracts of *F. tesmannii*, which probably helped in the inhibition of cell sickling by rehydrating red cells. More than 50% of sickled erythrocytes were reverted at 180 min during that study. Studied plant-derived drug has been demonstrated to contain principles that possess the ability to facilitate the stability of biological membranes when exposed to induced lysis [46]. Several reports have supported the fact that the membranes of human erythrocytes HbSS blood types have varied stability as determined from the mean corpuscular fragility [34, 47]. Therefore, plant extracts that can positively affect the red cell membrane would be useful in sickle cell disease management. Literature review conducted during this study has proven that the harvested medicinal plants also contain important pharmacological effects and chemical compounds that are associated with a diversity of secondary metabolites with a predominance of polyphenols. It is well-known that many diseases such as cancer, sickle cell anemia, and others are linked to the production of a high amount of oxidative stress [48]. Polyphenols are sources of antioxidant compounds that inhibit the oxidative stress and therefore may have antisickling activity to treat sickle cell disease.

## 5. Conclusion

Like in other studies cited in the body of text, this study successfully identified a diversity of medicinal plants that were commonly used by traditional healers in the treatment of sickle cell patients across some localities in the west region of Cameroon. Traditional healers used bark, seeds, and leaves that they administered through maceration, decoction, and chewing. Those plants were found to possess pharmacological effects and phytochemical compounds dominated by polyphenols and alkaloids, which are a great source of antioxidant activity. This finding provides some important information that can be used as a basis for future pharmacological studies to evaluate the therapeutic efficacy and safety of these promising medicinal plants. Such studies may be able to scientifically validate antisickling properties of those medicinal plants under in vitro and in vivo (clinical trials).

## Figures and Tables

**Table 1:** Scientific names of the harvested plants.

Common names	Scientific names	Families	Part used	Reference numbers
Avocatier	*Persea americana* Mill	Lauraceae	Seeds	57756/HNC
Kouoptche	*Harungana madagascariensis* Lam. exPoir.	Clusiaceae	Leaves	39947/HNC
Yam afzeliaa	*Ficus thonningii* Blume	Moraceae	Leaves	44042/HNC
Chrysanthe	*Chrysanthellum americanum* (L.) Vatke	Asteraceae	Leaves	42400/HNC
Djansang	*Ricinodendron heudelotii* (Baill) Pierre ex Heckel	Euphorbiaceae	Bark	19695/SRF Cam
Jouon	*Bridelia micrantha* (Hochst) Baill	Phyllanthaceae	Bark	52652/HNC
Viande de biche	*Lannea kerstingii* Engl K. Krauss	Anacardiaceae	Bark	41675/HNC
Pion d'inde	*Jatropha curcas*	*Euphorbiaceae*	Leaves	33592/HNC
Faux manioc	*Jatropha gossypiifolia* Linn.	Euphorbiaceae	Leaves	25715/SFR Cam
*Cajanus*	*Piptadeniastrum africanum* (Hook. F.) Brenan	Mimosaceae	Seeds	49168/HNC
Bolongo	*Fagara tessmannii* Engl	*Rutaceae*	Bark	38960/HNC
Curcuma	*Curcuma longa*	Zingiberaceae	Rhizomes	42173/HNC

HNC: National Herbarium of Cameroon; SFR: Forest Reserve Company.

**Table 2:** Previous data on the studied plants.

Scientific names	Part used	Major phytochemical compounds	Therapeutic/pharmacological effects
*Persea americana* Mill	Seeds	Alkaloids, terpenoids and steroids, saponins, tannin, flavonoids, glycosides, tannins [17]	Diabetes, ulcer, gastric, endometriosis, hepatoprotective and renal properties, antioxidant, antibacterial activities, hypoglycemia, antiviral activities, analgesic and anti-inflammatory activity, effect on body weight, vasorelaxant activity [18]
*Harungana madagascariensis* Lam. exPoir.	Leaves	Alkaloids, saponins, flavonoids, anthrones, anthraquinones, xanthones, essential oils [19, 20].	Malaria, liver blindness, ulcers, asthma, hepatitis, dysmenorrhea, toothache, chest pain, urogenital infections
*Ficus thonningii* Blume	Leaves	Alkaloids, polyphenols, tannins, saponosides, flavonoids [21]	Antiprotozoal, antifungal, antihelmintic properties, analgesic effects, cardioprotective effects, hypoglycemic effects, antidiarrheal effects, acute, sub-chronic, chronic and cytotoxicity [22–24]
*Chrysanthellum americanum*(L.) Vatke	Leaves	Tannins, saponosides, flavonoids	Liver protection, antioxidant properties, antihypertensive activities [25]
*Ricinodendron heudelotii* (Baill) Pierre ex Heckel	Bark	Tannins, saponins, flavonoids, alkaloids, carotenoids, phenols, steroids, cardiac, glucoside, terpenoids [26]	Inflammatory and anticarcinogenic properties, antioxidant activities [27]
*Bridelia micrantha* (Hochst) Baill	Bark	Flavonoids, polyphenolic compounds	—
*Lannea kerstingii* Engl K. Krauss	Bark	—	Antibacterial and anticonvulsant activity, cytotoxicity and antiproliferative effects [28]
*Jatropha curcas*	Leaves	Alkaloids, glycosides, flavonoids, saponins, tannins and terpenoids	Mouth infections, anticancerous properties, skin diseases, sores, muscular pain, malaria, antibiotic activity, buttons [29]
*Jatropha gossypiifolia* Linn	Leaves	Alkaloids, glycosides, flavonoids, saponins, tannins and terpenoids	Antihypertensive action, antimicrobial action, anti-inflammatory and analgesic action, healing action, antianemic, anticancer, malaria [30]
*Piptadeniastrum africanum* (Hook. F.) Brenan	Seeds	Flavonoids, tannins, alkaloids, saponins, cyanogenic glycosides, glycosides, and anthocyanins [31]	
*Fagara tesmannii* Engl	Bark	Alkaloids, lignans, phenols, amide, acidic phenol, coumarins, saponosides, flavonoids [32]	Spermatogenesis, testosterone level and sperm transit, decreased body weight, decreased insulin resistance and hyperglycemia [33], antibacterial activities [34]
*Curcuma longa*	Rhizomes	Triterpenes, flavonoids, phenols, anthraquinones, saponins, anthrocyanines [35]	Antioxidant, antineoplastic, anti-inflammatory, antimicrobial properties [36]; liver disease, jaundice, menstrual difficulties, hematuria, flatulence [37]

## Data Availability

Data can be obtained from the corresponding author.
